# Evaluation of metagenome binning: advances and challenges

**DOI:** 10.1093/bib/bbaf617

**Published:** 2025-11-21

**Authors:** Arangasamy Yazhini, Étienne Morice, Annika Jochheim, Benjamin Lieser, Johannes Söding

**Affiliations:** Quantitative and Computational Biology, Max-Planck Institute for Multidisciplinary Sciences, 37077 Göttingen, Germany; Quantitative and Computational Biology, Max-Planck Institute for Multidisciplinary Sciences, 37077 Göttingen, Germany; International Max-Planck Research School for Genome Sciences, University of Göttingen, 37077 Göttingen, Germany; Quantitative and Computational Biology, Max-Planck Institute for Multidisciplinary Sciences, 37077 Göttingen, Germany; International Max-Planck Research School for Genome Sciences, University of Göttingen, 37077 Göttingen, Germany; Quantitative and Computational Biology, Max-Planck Institute for Multidisciplinary Sciences, 37077 Göttingen, Germany; Quantitative and Computational Biology, Max-Planck Institute for Multidisciplinary Sciences, 37077 Göttingen, Germany; Campus Institute Data Science (CIDAS), University of Göttingen, 37077 Göttingen, Germany

**Keywords:** metagenomics, binning benchmarking, MAGs, microbiome, deep learning binners

## Abstract

Several recent deep learning methods for metagenome binning claim improvements in the recovery of high-quality metagenome-assembled genomes. These methods differ in their approaches to learn the contig embeddings and to cluster them. Rapid advances in binning require rigorous benchmarking to evaluate the effectiveness of new methods. We have benchmarked newly developed state-of-the-art deep learning binners on CAMI2 and real metagenomic datasets. The results show that SemiBin2 and COMEBin give the best binning performance, although not always the best embedding accuracy. Interestingly, post-binning reassembly consistently improves the quality of low-coverage bins. We find that binning coassembled contigs with multi-sample coverage is effective for low-coverage dataset, while binning sample-wise assembled contigs with multi-sample coverage (multi-sample) is effective for high-coverage samples. In multi-sample binning, splitting the embedding space by sample before clustering showed enhanced performance compared with the standard approach of splitting final clusters by sample. Deep-learning binners using contrastive models emerged as the top-performing tools overall, with MetaBAT2 and GenomeFace demonstrating superior speed. To facilitate future development, we provide workflows for standardized benchmarking of metagenome binners.

## Introduction

Shotgun metagenomics is widely used to study the genetic diversity, taxonomic composition, and metabolic potential of microbial communities. It allows the reconstruction of metagenome-assembled genomes (MAGs), which are valuable for building genomic catalogs, profiling genetic variations, studying accessory genes in strains, and analyzing functional repertoires of microbiomes [1–9]. Since many microbes lack close relatives in reference databases, MAG reconstruction requires an unsupervised approach [10]. Our study focuses on metagenome binning, a key step in reconstructing MAGs without recourse to any reference genomes.

**Figure 1:**
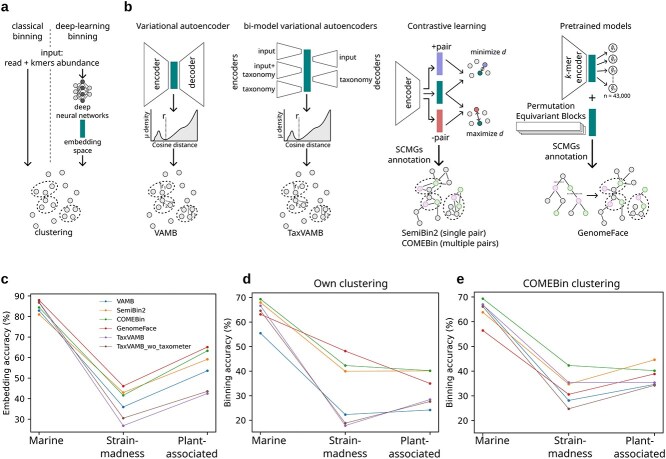
Overview of deep learning binning tools and their accuracy on gold standard coassembled contigs. a, b Schematic overview of a classical metagenome binning approach (a) and neural network architectures used in deep learning-based binning methods (b). In b, the symbol *d* represents the distance between the embedding vectors of an augmented contig pair. The cyan box indicates the feature embedding space learned by the neural network, which is used for clustering contigs. c, Cross-validated quality of embeddings quantified as the average accuracy of a multi-class linear classifier on the validation datasets. d, e Accuracy of bins produced after clustering evaluated using AMBER [35]. d, Accuracy of bins generated by each binner’s own clustering algorithm and e, accuracy of the same embeddings clustered using COMEBin’s iterative Leiden community detection algorithm, guided by SCMGs.

Metagenome binning is the process of clustering contigs assembled from shotgun reads based on their putative genomic origin, resulting in MAGs. The quality of MAGs is often lower than reported values, highlighting the need for further improvements in binning [11, 12]. Metagenome binning clusters the contigs, which are represented as a vector of two types of information: (i) *k*-mer frequencies (typically tetramers), which are similar within the same genome but differ across genomes from different genera. This feature is effective for distinguishing contigs at the genus-level but not at the species or strain level, and (ii) contig coverage in the sample, which is the average number of aligned reads per position, correlating with genome abundance (Fig. 1a). If multiple samples are available, this information source is very valuable because contigs from the same genome will exhibit similar coverage profiles across samples, providing complementary information for distinguishing contigs at the species level [13]. In addition to these two primary sources, some tools integrate taxonomic annotations [14–16] and assembly graphs, which represent links between contigs in the de Bruijn graph [17, 18]. For a comprehensive overview, we refer readers to a recent review by Mallawaarachchi *et al*. [19]. We also highlight pipelines Anvi’o [20] and EasyMetagenome [21] for users seeking streamlined, integrated workflows for read processing, binning, and downstream metagenome analysis.

State-of-the-art binners train deep networks to learn lower dimensional embeddings from the input coverage and *k*-mer frequency vectors of each contig, and perform clustering in the embedding space (Fig. 1a). For example, the first deep learning binner, VAMB [22], trains a variational autoencoder to learn the embedding. Its recent extension, TaxVAMB [16], integrates taxonomic annotations with coverage and *k*-mer frequency inputs and trains bi-modal variational autoencoders to learn the embedding space (Fig. 1b).

SemiBin [15] and SemiBin2 [23] introduced contrastive learning to train the network to keep similar contig pairs close together, while dissimilar pairs are placed far apart in the embedding space. To generate contig pairs, data augmentation was used, which involves splitting contigs into shorter fragments and recomputing their coverage and $k$-mer frequencies [14]. SemiBin also provided 11 pretrained models tailored to specific environments, such as human gut and soil, that can be used to infer the contig embedding space without training. COMEBin [24] extended contrastive learning by using more augmented pairs and processing coverage and *k*-mer data separately. In a novel approach, GenomeFace [25] infers the embedding space directly from the input contig vectors using two pretrained networks. One is trained on *k* mer frequencies with *k* values ranging from 1 to 10 using random contigs from 43 000 curated microbial genomes to predict their taxonomic label from the embeddings. Another network is a transformer model trained on read coverage data using permutation-invariant properties.

Besides variations in deep learning models, these tools differ in the clustering algorithms used to group contigs in the feature embedding space. VAMB uses iterative density-based clustering, while COMEBin uses the Leiden algorithm and GenomeFace uses hierarchical clustering on a geodesic minimum spanning tree constructed from the embedding space. COMEBin and GenomeFace optimize the clustering process based on measures of completeness and purity derived from the occurrence of single-copy marker genes (SCMGs) in the bins [26, 27]. SemiBin uses the Infomap algorithm [15] to generate bins and, if their mean number of SCMGs exceeds one, they are further re-clustered with weighted k-means based on embeddings and abundance.

Binning can be performed using one of the following three approaches: (i) coassembly multi-sample binning, where reads from multiple samples are pooled, coassembled, and binned using abundance across all samples; (ii) multi-sample binning, where reads are assembled per sample, contigs are binned collectively using multi-sample abundance, and bins are split by sample origin; and (iii) single-sample binning, where contigs are assembled per sample and binned using only their own sample’s abundance [28]. Each approach has trade-offs.

Coassembly multi-sample binning increases coverage, enabling the assembly of low-abundance genomes. However, pooling reads from multiple samples introduces higher strain diversity, leading to fragmented assemblies due to ambiguities in contig extension across conserved regions [29]. Single-sample binning is often used in large-scale metagenomics studies due to its computational efficiency [4, 30, 31]. While it reduces strain-diversity-related issues, it does not exploit multi-sample abundance data; also, it poorly recovers low-abundance species, and produces highly similar MAGs across samples, making dereplication essential. A prior study found that sample-wise assembly produces a larger nonredundant gene set than coassembly [29]. VAMB demonstrated that multi-sample binning yields more high-quality, strain-resolved bins than single-sample binning [22]. However, no study has compared the nonredundant results of single-sample and multi-sample binning with the results of coassembly multi-sample binning.

Rapid advances in metagenome binning and diverse sampling strategies underscore the need for a critical evaluation of state-of-the-art methods to assess their effectiveness in generating high-quality MAGs. In this study, we benchmark recent deep learning-based binning tools using three CAMI2 datasets of varying complexity in genome coverage, contig contiguity and strain diversity and three real metagenome datasets. Using gold-standard contigs, we independently evaluated the accuracy of the embedding space prior to clustering and the binning accuracy using either their own clustering algorithm or the same clustering algorithm. Using real-assembled contigs, we compared the number of bins meeting quality criteria produced by various deep learning methods. In addition, we present the first comparative analysis of metagenomic binning performance after post-binning reassembly and across the described three different binning approaches.

## Results

### Accuracy of feature embedding space and clustering in deep learning binners

Binning tools based on deep learning models typically operate in two phases: (i) learn an embedding in a lower dimensional space for each contig and (ii) clustering contigs represented by their embedding vectors into genomic bins (Fig. 1a). To compute an embedding function for each contig represented by a feature vector containing its abundances across various samples and its $k$-mer composition, typically across 4-mers, deep neural networks have gained popularity recently. Our first goal was to evaluate the accuracy of contig embeddings generated by different deep learning-based binners, independent of the ensuing clustering process. We evaluated five methods: VAMB (variation autoencoder), TaxVAMB (bi-modal variational autoencoders) with and without taxonomy refinement using Taxometer [32], SemiBin2 (contrastive learning using a single augmented pair per contig), COMEBin (contrastive learning using multiple augmented pairs per contig), and GenomeFace (pretrained models). Figure 1b summarizes the different learning objectives used to train the embedding neural networks. The performance of the binning tools was evaluated by binning gold standard contigs from the CAMI2 marine, strain-madness, and plant-associated datasets [33]. The datasets consist of simulated paired-end reads and coassembled contigs, labeled with their source genomes. Contigs were generated by selecting genomic regions from source genomes where simulated reads aligned with a coverage of at least 1 [34]. These datasets exhibit substantial variation in genome fraction covered by the simulated reads (26%–90%), contig contiguity (NGA50 ranging from 87 911 to 682 777 bp), and strain diversity (81–395 strains).

To assess the accuracy of the embeddings, we trained a multi-class linear classifier with softmax output using the embedding feature vectors as input to predict the correct genome, and we evaluated its accuracy through five-fold cross-validation (Methods). Classification accuracy was computed as the ratio of correctly classified contigs to the total, based on ground truth labels. Figure 1c reports the embedding accuracy of the five tools in three CAMI2 datasets. The average accuracy was 85% for the marine dataset, 37.3% for the strain-madness dataset, and 54.5% for the plant-associated dataset, suggesting that the embedding accuracy is strongly influenced by the dataset complexity. For the marine dataset, GenomeFace achieved the highest embedding accuracy (88%), followed by TaxVAMB (87%), COMEBin (84%), and VAMB (82.9%). For the strain-associated dataset, GenomeFace outperformed the others with an accuracy of 46.1%, followed by SemiBin2 (43%), COMEBin (41.6%), and VAMB (35.9%). For the plant-associated dataset, GenomeFace again achieved the highest accuracy (65.1%), followed by COMEBin (63.3%), SemiBin2 (59.1%), and VAMB (53.6%). Overall, GenomeFace was the most effective tool for embedding accuracy across the three datasets. The superior performance of GenomeFace observed in this evaluation is attributed to the composition neural network, which was trained as a classifier to predict genomic labels of contigs with softmax cross-entropy loss.

We evaluated the accuracy of the bins obtained after clustering the embedding space using AMBER [35]. AMBER’s binning accuracy is defined as the average proportion of correctly clustered base pairs into assigned genomic bins (true positives) relative to the total base pairs binned. Figure 1d shows the binning accuracy of five binners. The average values for the marine, strain-madness, and plant-associated datasets were 64.5%, 31.6%, and 32.6%, respectively. This trend reflects the accuracy of the embedding space and highlights the challenges posed by the strain- and plant-associated datasets, particularly due to high fragmentation (Methods). COMEBin achieved the highest binning accuracy for the marine (69.3%) and plant-associated (40.2%) datasets. GenomeFace showed the best binning accuracy for the strain-madness dataset (48.2%). In Supplementary Fig. 1, we compared average bin purity and bin completeness. Bin purity is calculated as the proportion of base pairs in a bin that overlap with the assigned genome. Bin completeness is calculated as the proportion of base pairs of the genome covered by the assigned bin. VAMB and TaxVAMB achieved the best average purity for the marine dataset (95.1%), while GenomeFace achieved the best average purity for the strain-madness (78%) and plant-associated (96.5%) datasets. COMEBin achieved the highest average completeness for the marine dataset (64.9%), while VAMB showed the highest completeness for the strain-madness (75.1%), and SemiBin2 showed the highest completeness for plant-associated (22.2%) datasets.

For the clustering, SemiBin2, COMEBin, and GenomeFace use SCMGs to refine bins, whereas VAMB and TaxVAMB do not. To evaluate the binning accuracy of the feature embedding spaces learned by each tool using the same clustering procedure, we applied COMEBin clustering to all embeddings and evaluated the resulting bins using AMBER. We selected COMEBin clustering because it is the most comprehensive, leveraging the largest set of SCMGs and an iterative Leiden algorithm across 120 parameter combinations. In Fig. 1e shows the binning accuracy using COMEBin clustering. COMEBin remained the top performer on two of the three datasets analyzed, while SemiBin2 achieved the highest binning accuracy on the plant-associated dataset. The average binning accuracy across tools is increased by 0.4% for marine (binning accuracy: 64.9%), 1% for strain-madness (32.6%) and 5.4% for plant-associated (38%) datasets. An increase in binning accuracy with COMEBin clustering was consistently observed for VAMB and TaxVAMB (Supplementary Table 1). In contrast, accuracy decreased for SemiBin2 and GenomeFace in two out of three datasets, suggesting that COMEBin clustering primarily benefits binners that do not already incorporate SCMGs in their default clustering strategy. The reduced performance of COMEBin clustering on embeddings from other tools illustrates that finding clustering methods that align well with the embedding characteristics is essential to achieve optimal binning accuracy.

### Benchmarking binning performance on semisynthetic and real coassembled contigs

**Figure 2:**
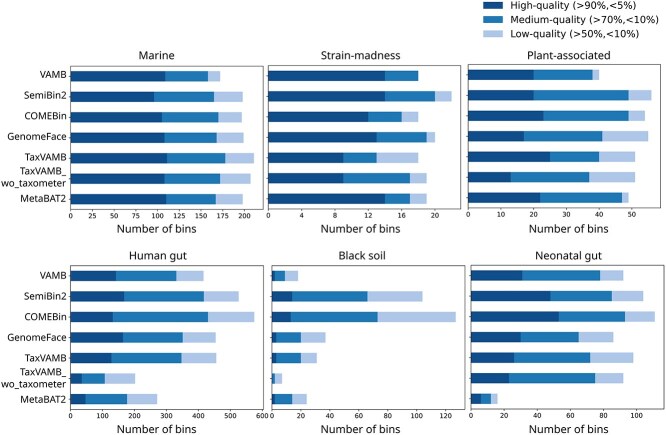
Binning performance on semisynthetic and real coassembled contigs with multi-sample coverage. The number of high-quality (90% completeness and 5% contamination), medium-quality (70% completeness and 10% contamination), and low-quality (50% completeness and 10% contamination) bins generated by different binners for six datasets, as predicted by CheckM2 [37].

We benchmarked the binning performance of deep learning methods on real metagenomic contigs by coassembling reads pooled across all samples using MEGAHIT [36]. For this analysis, we used the three CAMI2 datasets and three real datasets. We evaluated the binners using CheckM2 [37], which estimates the completeness and contamination of each bin through neural network models, providing results comparable with those of AMBER (Supplementary Materials Figs 2 and 3). We performed binning of these coassembled contigs using read coverage from all metagenomic samples (coassembly multi-sample). To compare the binning performance of deep learning-based methods with a widely used traditional approach, we used results from MetaBAT2. It has consistently outperformed other traditional binning tools and shown competitive performance with deep learning tools in previous publications [15, 22, 24, 38]. We therefore selected it as the baseline for comparison in our study. We evaluated the number of high-, medium-, and low-quality bins produced by each tool. Figure 2 reports the number of bins produced across the three quality categories for six metagenome datasets. For the marine dataset, TaxVAMB achieved the highest number of bins (111 high-quality, 178 medium-quality, and 211 low-quality), with only small differences compared with the numbers produced by other binning tools. SemiBin2 achieved the highest number of bins for strain-madness (14, 20, and 22) and plant-associated datasets (20, 49, and 56).

For the real datasets, COMEBin yielded the highest number of bins for human gut samples (132 high-quality, 430 medium-quality, and 575 low-quality bins), black soil (13, 73, and 127), and neonatal gut samples (53, 93, and 111). Compared with CAMI2 datasets, the total number of bins produced by each tool for real datasets is more varied, with SemiBin2 being the second-best tool. These results suggest that binning using contrastive models outperform other neural network models for real datasets. Using Taxometer [32] in TaxVAMB for taxonomy refinement substantially improves the yield of high-quality bins compared with TaxVAMB without refinement (111 versus 108 for marine, 25 versus 13 for plant-associated, 128 versus 35 for human gut, 3 versus 0 for black soil, and 26 versus 23 for neonatal gut). For strain-madness, there was no difference in the number high-quality bins in TaxVAMB with and without taxonomy refinement. Furthermore, TaxVAMB (nine bins) produced fewer high-quality bins than VAMB (14 bins), indicating that VAMB is more suitable for strain-rich datasets. Overall, the deep-learning binners outperformed the widely used, traditional non-deep learning tool MetaBAT2. Although the difference in the CAMI2 datasets is small, deep-learning binners significantly outperform MetaBAT2 on real datasets. Among deep learning methods, GenomeFace outperforms TaxVAMB on four datasets including human gut and black soil datasets, despite not using dataset-specific training. This highlights the potential of developing or utilizing pretrained models for metagenomic binning.

### Post-binning reassembly benefits low-coverage bins

**Figure 3:**
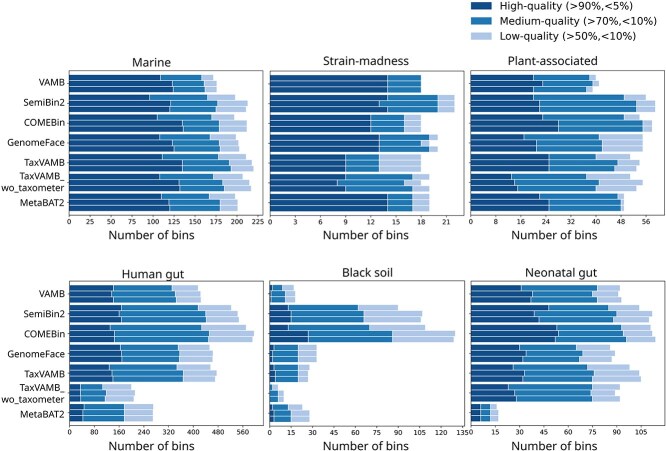
Evaluation of reassembly after binning. For each dataset and tool, the top bars indicate the number of bins categorized into high-, medium-, and low-quality groups before reassembly, the middle bars show bin counts after reassembly, and the bottom bars represent bin counts after reassembling only low-coverage bins (mean bin coverage $\leq $ 100).

MetaWRAP has demonstrated that reassembly after metagenome binning can improve bin quality [39]. We evaluated the effect of reassembly, using CheckM2, on genomic bins derived from coassembly multi-sample binning. In Fig. 3, the top bars correspond to the number of bins in three quality categories without reassembly and the middle bars correspond to the number of bins after reassembly of all bins. On average, reassembly produced 20 additional high-quality bins in the marine dataset, three more in the plant-associated dataset, and two more in both the black soil and neonatal gut datasets. No increase in high-quality bins was observed for the strain-madness dataset, while gains in medium- and low-quality bins were noted for the human gut samples. We investigated whether bin coverage is associated with the improvements in bin quality upon reassembly. Supplementary Fig. 4 shows that reassembly substantially improved completeness and marginally improved purity, but only for low-abundance bins. In contrast, reassembly was either ineffective or detrimental to the completeness and/or purity of bins with high read coverage across datasets. To test whether the observed improvements were primarily driven by low-coverage bins, we performed reassembly exclusively on bins with coverage<100. This selective reassembly yielded improvements in the number of bins across the three quality categories (bottom bars, Fig. 3) comparable with those obtained by reassembling all bins, and a clear improvement over no reassembly (Fig. 3, top bars).

Furthermore, improvements by reassembly differed between the three bin quality categories (Supplementary Fig. 5). Reassembly primarily benefited the high-quality bins (mean increase across five datasets is 11.6%) followed by the medium-quality bins (11.2%), and had the least positive effect on the low-quality bins (4%) of all binning tools tested. For strain-madness, reassembly has a negative effect for all three quality category bins, implying that reassembly may not help in high-coverage, high strain-diversity datasets.

### Comparison of different binning approaches

**Figure 4:**
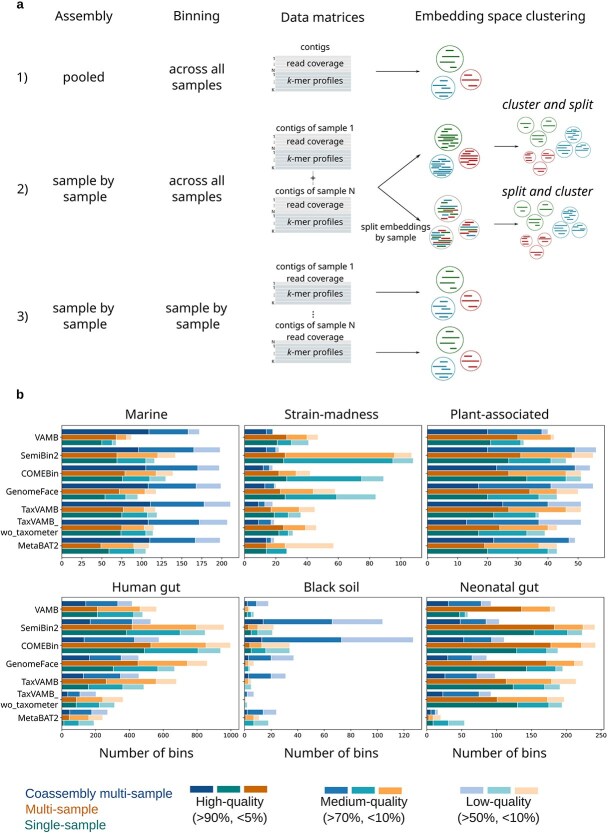
Comparison between different assembly and binning approaches. a, (1) Coassembly multi-sample, (2) multi-sample, and (3) single-sample. In deep learning methods, multi-sample binning can be performed either by clustering contigs in the embedding space and then splitting clusters by sample (cluster and split), or by splitting contigs in the embedding space by sample and then clustering each sample independently (split and cluster). b, The number of high-, medium-, and low-quality bins obtained from coassembly multi-sample, multi-sample, and single-sample binning. Completeness and purity were obtained from CheckM2 evaluation [37].

As illustrated in Fig. 4a, metagenome binning can be performed using different approaches: (1) assembly of pooled reads followed by multi-sample binning (coassembly multi-sample), (2) sample-wise assembly followed by multi-sample binning (multi-sample), and (3) sample-wise assembly and binning (single-sample). In the coassembly multi-sample approach, reads from all metagenomic samples are assembled together and the read coverage of all samples is used to bin contigs. This approach was employed to obtain the results presented in previous sections. The multi-sample approach bins contigs assembled separately for each sample using read coverage data from all samples. The single-sample approach involves both assembly and binning performed independently for each sample. We compared binning performance across the three approaches based on the total number of high-, medium-, and low-quality bins, as evaluated by CheckM2. We performed dereplication to remove redundancy in the multi-sample and single-sample binning results, and then compared them with the coassembly multi-sample binning results (Methods).

Multi-sample binning in deep-learning tools can be implemented in two ways: (i) using multi-sample read coverage to bin contigs independently per sample (e.g. SemiBin2), or (ii) binning contigs from all samples together with multi-sample coverage and then splitting the final bins by sample (e.g. VAMB) (Fig. 4a). In the latter strategy, sample-wise splitting is performed either before clustering (“split and cluster,” as in GenomeFace) or after clustering (“cluster and split,” as in VAMB, COMEBin, and TaxVAMB). First, we tested these two modes only in VAMB, COMEBin, and TaxVAMB, where both modes were possible. Supplementary Fig. 6 showed that on average across tools and datasets, split and cluster mode produced 19% more bins, including 79% more high-quality bins, compared with cluster and split mode. Therefore, we used binning results of the split-and-cluster mode to compare with other binning approaches. An exception was observed in the COMEBin output for the neonatal gut dataset, where the cluster and split mode (178 high-quality and 242 total bins) marginally outperformed the split and cluster mode (173 and 241 bins), and thus was used in the comparison with other binning approaches.

Among the three binning approaches compared in terms of the total number of bins in three-quality categories (Fig. 4b), coassembly multi-sample binning performed the best for the marine, plant-associated, and black soil datasets. It produced on average 79, 3, and 38 more bins than multi-sample binning, 91, 9, and 37 more bins than single-sample binning in the marine, plant-associated, and black soil datasets, respectively.

For the remaining datasets, multi-sample binning generally achieved the highest binning performance across tools. On average, it produced 38, 251, and 103 more bins than coassembly multi-sample binning for strain-madness, human gut, and neonatal gut datasets, respectively. Compared with single-sample binning, multi-sample binning produced on average 106 and 31 more bins for human gut and neonatal gut datasets, respectively. For the strain-madness dataset, multi-sample binning yielded, on average, 15 more bins than single-sample binning across three binning tools. However, single-sample binning produced 25 more bins than multi-sample binning for results specifically from SemiBin2, COMEBin2, and GenomeFace.

To investigate whether read coverage influences binning performance, we compared the mean total read coverage of binned contigs across datasets (Supplementary Fig. 7). We observed that marine (14.6), black soil (15.4), and plant-associated (43.7) datasets are low to moderate coverage, while the human gut (84.9), neonatal gut (131.3), and strain-madness (1662.1) are high coverage. These results suggest that coassembly multi-sample binning is particularly effective for low or moderate coverage datasets, whereas multi-sample binning performs better for datasets with high coverage. Single-sample binning may be advantageous for strain-rich datasets with high read coverage, such as strain-madness.

### Run time and memory usage

We evaluated the run time and peak memory usage of binners for coassembly multi-sample binning in six datasets. GenomeFace, and MetaBAT2 can directly utilize abundance data in tabular format, whereas VAMB, COMEBin, and TaxVAMB require BAM alignment files to extract abundance information, significantly increasing run time. Therefore, we compared the runtime of tools for which we input BAM files for abundance data, with and without preprocessing of alignment files. We considered data augmentation as a part of the preprocessing in SemiBin2 and COMEBin.

**Figure 5:**
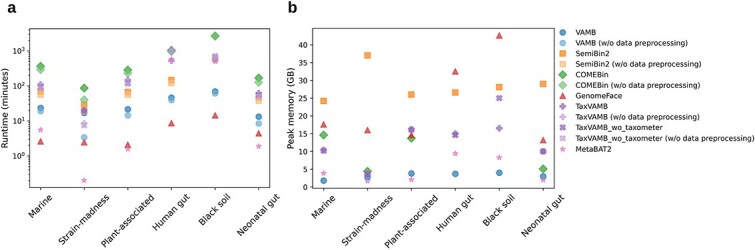
Computational efficiency. a, Run time (in minutes) required by state-of-the-art deep learning tools for binning. The y-axis is plotted on a logarithmic scale. b, Peak memory usage (in gigabytes) observed during the execution of each binning tool.

Run time comparison in logarithmic scale of minutes in Fig. 5a reports that MetaBAT2 was the fastest tool for strain-madness (0.2 min), plant-associated (1.6 min), and neonatal gut (1.9 min) datasets, $\sim $12x, 1.4x, and 2.6x faster, respectively, than the second fastest tool, GenomeFace. For the marine, human gut, and black soil datasets, GenomeFace was the fastest, completing runs in 2.6, 8.6, and 14.4 min, which are 2$\times $, 5$\times $, and 4.5$\times $ times faster than the second fastest tools. However, GenomeFace required additional processing time to identify SCMGs on a single CPU node: 60 min for marine, 6.6 for strain-madness, 39 for plant-associated, 50.5 for human gut, 31.3 for black soil, and 15 min for neonatal gut datasets. Among different VAMB extensions, the original VAMB runs on average 6$\times $ faster than TaxVAMB and 8$\times $ faster when preprocessing is excluded. SemiBin2 also outperformed TaxVAMB in speed for the plant-associated (2$\times $ faster) and human gut (3.6$\times $ faster) datasets, with comparable performance for others. COMEBin was the slowest tool across all datasets. When preprocessing time is excluded, it remains the slowest, except for the human gut dataset. While all tools showed increased runtime with a growing number of contigs, MetaBAT2 demonstrated a steeper increase compared with the deep learning-based methods.

Figure 5b shows that VAMB had the lowest peak memory usage for the marine (1.8 GB), human gut (3.7 GB), and black soil (4 GB) datasets. This is half the peak memory usage of the second best tool, MetaBAT2. For the remaining datasets, MetaBAT2 had the lowest peak memory usage: 1.7 GB (strain-madness), 2.1 GB (plant-associated), and 2 GB (neonatal gut), averaging 1.6 times lower than VAMB. Across six datasets, TaxVAMB used on average 3.8 times more memory than VAMB. COMEBin had lower peak memory usage than SemiBin2 and GenomeFace on four datasets. COMEBin runs on the human gut and black soil datasets were terminated after exceeding the 5-day cluster runtime limit due to inefficient parallelization of its clustering procedure. As a result, peak memory usage could not be recorded for those two datasets. SemiBin2 had the highest peak memory usage for marine (24.2 GB), strain-madness (37 GB), plant-associated (26 GB), and neonatal gut (29 GB) datasets, while GenomeFace showed the highest peak memory usage on the human gut (32.5 GB) and black soil (42.6 GB) datasets.

## Discussion and outlook

Metagenome binning is a challenging clustering problem that has seen considerable progress with advances in representation learning. Recent benchmarking efforts have provided a comprehensive evaluation of binning tools and their performance on real datasets [38]. In this study, we provide complementary benchmarking results for metagenome binning. Our work differs from the CAMI2 challenge [33] and the recent study by Han *et al.,* (2025) [38], in five key aspects: (i) we compared recently developed state-of-the-art deep learning binners based on diverse underlying methodologies, (ii) we independently assessed the accuracy of the learned embeddings and the clustering, (iii) we evaluated binning performances on both simulated and real metagenome datasets, (iv) we investigated the impact of reassembly after binning, and (v) we evaluated the performance of co-assembly multi-sample, multi-sample, and single-sample binning approaches.

Among the methods tested, the two contrastive learning-based binners SemiBin2 and COMEBin achieved the highest binning performance, surpassing the best results of a single binner in the CAMI2 challenge [33](Figs 2 and 4b). They showed clear advantage over other deep-learning approaches, as they use more information through data augmentation and explicitly training to keep contigs from the same genome proximal and contigs from different genomes far apart in the feature embedding space. VAMB and TaxVAMB rely on neural networks for nonlinear dimensionality reduction of input features, which appears less effective than contrastive learning on complex datasets such as human gut and soil samples (Figs 2 and 4b).

GenomeFace showed lower performance compared with contrastive learning-based models. As it is trained solely on known reference genomes, its effectiveness appears to be limited in understudied environments. Nonetheless, due to its speed and better performance than the variational autoencoder-based methods (Figs 2, 4b and 5), GenomeFace is a promising binning tool for large-scale real-world metagenomic datasets. Notably, all deep learning-based methods outperformed the classical binner MetaBAT2 on real datasets, underscoring the importance of denoising input features before clustering to enhance binning results.

Reassembly has been proposed as a post-binning refinement approach to improve MAGs recovery [39–41]. Our results indicate that the benefit of reassembly depends on the read coverage of the bins. It significantly improves the completeness and marginally improves purity for low-coverage bins, while providing no benefit or detriment for high-coverage bins in all six datasets (Fig. 3). This may be due challenges in initial coassembly. To decide which paths through the de-Bruijn graph to extract as contigs, assemblers try to find the paths with high and consistent coverage. Paths/contigs with low coverage are much harder to distinguish from other paths/contigs with similar low coverage. During reassembly, only a small subset of reads is assembled, resulting in much simpler de-Bruijn graphs with fewer ambiguities. In contrast, high-coverage reads are already well assembled during the initial co-assembly, making reassembly less beneficial in such cases. Therefore, the decision to use reassembly should be guided by bin coverage.

We find that the available reassembly module from MetaWRAP has limitations: it does not support interleaved reads, and does not handle reads from multiple samples efficiently, which can lead to memory constraints. To address these issues, we provide a simple reassembly workflow that bypasses MetaWRAP’s read mapping step for reassembly (Supplementary Fig. 11). Our workflow also parallelizes reassembly across bins, improving time efficiency.

Mattock and Watson [28] compared multi-sample binning with single-sample binning and showed significant differences in bin yield. However, this study did not include the widely used coassembly multi-sample binning approach for benchmarking studies. We performed an extensive comparison of three different binning approaches and report that, given the multi-sample coverage data, the binning of coassembled contigs outperformed multi-sample contigs for the low-coverage samples (marine, plant-associated, and black soil datasets in Fig. 4). In the marine dataset, coassembly produces longer contigs, resulting in more reliable read coverage and $k$-mer profiles and better binning results (Supplementary Fig. 8). For black soil dataset, where both approaches generate contigs of similar lengths, coassembly multi-sample binning remains more effective. We speculate that this is due to the same genomic region being represented by several contig from different samples. When reads are mapped to a concatenated set of contigs, they are assigned to only one of these contigs, resulting in reduced read counts. In contrast, in coassembly multi-sample binning, genomic regions are likely represented by a single contig, allowing unambiguous read mapping. This results in higher counts, more accurate abundance profiles, and thus better binning performance.

In the human gut and neonatal gut datasets, contigs in coassembly multi-sample binning are shorter than contigs in multi-sample binning due to high fragmentation in the coassembly (Supplementary Fig. 8). Both datasets are high abundant samples. Current genome assemblers struggle to expand assemblies when reads from other abundant samples belonging to different genomes share highly similar regions. By assembling reads of each sample independently, multi-sample binning avoids fragmentation due to ambiguities in possible extensions. Longer contigs have more read counts and less noisy abundance profiles for binning. Thus, multi-sample binning is advantageous for datasets with high per-sample coverage datasets. We recommend the “split and cluster” mode, which improves binning results compared with the default “cluster and split” mode in existing multi-sample binning tools (Supplementary Fig. 6). Additionally, dereplication by selecting the best bin within each genomic cluster across samples may result in the loss of valuable genomic information contained in unselected bins from other samples. Developing an efficient method to merge bins across samples could further enhance the performance of multi-sample binning.

Run time evaluation showed that MetaBAT2 and GenomeFace were the fastest tools. COMEBin was the slowest tool, taking up to 2 days for multi-sample binning and causing out-of-memory problems during its clustering step. Addressing these limitations could make COMEBin more suitable for large-scale metagenomic studies. In terms of memory usage, although SemiBin2 and GenomeFace show the highest peak memory requirement (Fig. 5b), they remained within the RAM limits (e.g. 64 GB) of modern computers.

A limitation of this benchmarking is its focus on prokaryotic genomes only, as metagenome binning tools and evaluation methods are standardized for prokaryotes (bacteria and archaea). For example, SCMGs used in supervised clustering for binning, and training datasets for evaluation tools such as CheckM2 [37] and DeepCheck [42], contain predominantly prokaryotic and archaeal genomes. Although TaxVAMB did not show the best performance in this benchmarking study, it has, however, demonstrated improvements in binning both prokaryotic and fungal genomes by incorporating comprehensive taxonomic data [16]. In addition, genome foundation models such as DNABERT-S [43] have improved the accuracy of sequence composition embeddings. With rapid taxonomic annotation tools such as MMseqs2 taxonomy [44], Metabuli [45], and Taxometer [32], future binners combining genome foundation models with taxonomic data in semi- or self-supervised multimodal approaches could improve genome binning across diverse domains of life.

In summary, our study demonstrates that while the yield of MAGs is largely influenced by dataset complexity, the choice of assembly and binning strategies plays a crucial role in optimizing MAG recovery. For high-complexity datasets with many genomes of low coverages, we recommend coassembly multi-sample binning. For high-coverage datasets, multi-sample is the most effective approach, while single-sample binning is advantageous in strain-rich samples. To further enhance binning performance, we recommend performing post-binning reassembly particularly for low-coverage bins. For a graphical summary of these recommendations, see Supplementary Fig. 12.

## Methods

### Benchmarking datasets and assembly

We used the gold standard datasets from the CAMI2 assessment study [33], namely marine, strain-madness, and plant-associated samples. These datasets are recent, have different strain diversity, and are not widely used in the development of binning tools. The marine dataset contains 777 genomes (474 unique and 303 strains) with simulated reads for 10 samples. The strain-madness dataset contains 408 genomes (13 unique and 395 strains) with simulated reads for 100 samples. The plant-associated dataset contains 495 genomes (414 unique and 81 strains) with simulated reads for 21 samples. The gold standard coassembled contigs provided by the CAMI2 study show greater variability in genome coverage and sequence contiguity across the datasets. For marine, the contigs show moderate genome fraction coverage (76.9%) but are contiguous (NGA50 of 682 777 bp). The contigs of the strain-madness dataset show 90.0% genome fraction coverage but low contiguity (NGA50 of 155 980 bp). The plant-associated contigs have the lowest genome fraction coverage (29.6%) and are more fragmented (NGA50 of 87 911 bp) than the other two contig sets.

We found that the simulated reads had an error rate of 3%, significantly higher than the typical error rate of $0.1$ to $1\%$ for standard Illumina short reads [46]. To address this, we used CoCo (git@github.com:soedinglab/CoCo.git), an error correction tool that corrects reads based on *k*-mer counts. Since $k$-mer counts from pooled samples provide more accurate information than single sample data, we corrected reads by counting $k$-mers across all samples together. This error correction reduced the error rate from 3% to 1%. Coassembly was performed with MEGAHIT (v1.2.9) [36] using the --presets meta-sensitive option. For individual sample assemblies, corrected reads were separated by sample and independently assembled. We evaluated the assembled contigs by MetaQuast [47] and found that coassembled contigs cover 44.99%, 8.4%, and 14.1% fractions of the genomes for the marine, strain-madness, and plant-associated datasets, respectively, while contigs from sample-wise assembly cover 41.1%, 64.7%, and 8.8%. The mean lengths of contigs ($\geq $1000 bp) subjected to binning are 3557, 6018, and 3972 bp for coassembly, while the same for the multi-sample contigs are 3355, 4039, and 4920 bp for the three datasets, respectively (Supplementary Fig. 8).

### Real datasets and assembly

To evaluate the binning performance on real datasets, we used samples from three metagenomic studies: 15 gut samples from villagers in Honduras (BioProject accession: PRJNA999635) [48], 18 samples from black soil (BioProject accession: PRJNA1226397) [49], and 43 neonatal gut samples at day 21 postpartum (ENA Study Accession: ERP115334) [31]. The details of read processing and mapping are provided in [50]. Briefly, raw reads from gut samples were decontaminated using KneadData (v0.12.0) [51], while reads from black soil were error corrected using Musket (v1.1) [52]. For the coassembly, reads from each dataset were coassembled using MEGAHIT (v1.2.9) [36] with --presets meta-sensitive option. For the sample-wise assembly, reads from human gut and black soil samples were assembled using metaSPAdes (v4.0.0; *–only-assembler* and *-m 1000* settings). We used MEGAHIT (v1.2.9) to perform sample-wise assembly for neonatal gut samples due to the high computational demands of metaSPAdes. The mean lengths of coassembled contigs used for binning are 3179, 1864, and 4508 bp for the human gut, black soil, and neonatal gut datasets, respectively. For multi-sample binning, the mean contig lengths are 3830, 1715, and 8183 bp for the same datasets (Supplementary Fig. 8).

### Metagenome binning benchmark

To perform binning, we generated an abundance matrix by mapping reads to assembled contigs. Strobealign (v0.13.0-25-g3a97f6b) [53] was used to estimate abundance values using the --aemb option and to align reads from each sample to contigs to obtain alignment SAM files. GenomeFace and MetaBAT2 accept the abundance matrix directly, while VAMB, COMEBin, and TaxVAMB require sorted BAM files as input. We consolidated the abundance values from each sample into a matrix format (similar to MetaBAT2’s jgi_summarize_bam_contig_depths output) using an in-house Python script. Samtools (v1.19) [54] was used to create sorted BAM files from the alignments. For all binners, contigs of at least 1000 bp were used for binning, except for MetaBAT2, which accepts only contigs above 1500 bp. The commands used for the benchmarking runs are available in the Supplementary Materials.

For the TaxVAMB run, we used Metabuli [45] with the classify command and the --seq-mode 1 option to annotate taxonomy IDs to contigs, as it showed the best performance in TaxVAMB’s original benchmarking results [16]. We performed two separate TaxVAMB runs for all binning approaches: one using Taxometer [32] to refine taxonomy IDs mapped by Metabuli and another without refinement, using the --no_predictor option. Metabuli reports were processed using Taxconverter (https://github.com/RasmussenLab/taxconverter.git) to generate input taxonomy assignment file for TaxVAMB.

### Single-sample and multi-sample binning

We performed assembly for each sample independently. In single-sample binning, we binned sample-wise contigs using read coverage from only the corresponding sample. Binning was performed separately for each sample. For the SemiBin2 single-sample binning, we used pretrained models specific to each datasets: human_gut for the human gut and neonatal gut datasets, soil for the black soil dataset, and global for the three CAMI2 datasets.

In multi-sample binning, we binned contigs using read coverage from all samples. Contigs were concatenated from all samples and binned together. We performed multi-sample binning in cluster-and-split as well as split-and-cluster modes and evaluated results from VAMB, COMEBin, and TaxVAMB, as only these tools allowed the evaluation of two modes.

Single and multi-sample binning of the same environmental samples result in redundant bins due to the presence of the same genomes in multiple samples. To remove redundancy, we dereplicated bins using dRep [55] at the strain level (99% ANI) with 50% completeness and 10% contamination thresholds, and options --S algorithm skani, -sa 0.95, and -pa 0.99.

### Evaluation

To evaluate the accuracy of the embedding spaces generated by the binning tools, we used a linear multi-class classifier with softmax output implemented in PyTorch (v1.12.1), each genome being represented by one class. The classifier consists of a single linear layer with the following parameters: ‘learning rate’: 0.001, ‘optimizer’: Adam, ‘epoch’: 300, and ‘Loss’: cross entropy. For the evaluation, we used short gold-standard pooled contigs and corresponding genome labels for the marine, strain-madness, and plant-associated datasets from the CAMI2 study [33]. Each dataset was randomly divided into five groups for five-fold cross-validation using scikit-learn KFold [56]. We computed the classification accuracy as the ratio of correctly labeled contigs to the total number of contigs in the test dataset. The embedding space accuracy of the binning tool was defined as the average classification accuracy across the test datasets following five-fold cross-validation. This accuracy was then compared across five different binning tools.

We used AMBER [35] to evaluate the accuracy of metagenome binning and the quality of individual bins. Accuracy of binning is defined as the average of the number of base pairs that overlap with the genome assigned of the bin (TP) over the number of base pairs in the entire dataset, including base pairs not overlapping with the assigned genome (FP) and unassigned base pairs (TP+FP+Unassigned). We computed the average completeness (bp) and average purity (bp) of bins using AMBER [35]. Briefly, average purity (bp) is the fraction of correctly assigned base pairs in each bin, defined as the proportion of base pairs in a bin that belong to the predominant genome in that bin, averaged across all predicted bins generated by the binner. Average contamination is computed as 1 minus average purity. Average completeness (bp) refers to the fraction of base pairs from a genome that are covered by contigs within a given bin, averaged across the completeness values of all genomes in the sample. For bins generated from contigs assembled using MEGAHIT [36] or metaSPAdes [57], we used CheckM2 [37] to predict completeness and contamination. Bins were classified into three groups based on these two measures: high quality ($\geq $90% completeness and <5% contamination), medium quality ($\geq $70% completeness and $<10$% contamination), and low quality ($\geq $50% completeness and <10% contamination). In all cases, bins with a total length of at least 200 kb were selected for comparison.

### Post-binning reassembly

We used the SPAdes assembler (v4.0.0) [57] for reassembly with the --careful and --trusted-contigs flags. For each bin, reads were extracted from all samples using the ‘extractreads’ C++ executable with unsorted sam or bam input files. Reassembly was performed using the bin-specific reads together with the contigs belonging to that bin. Final scaffolds were filtered by a minimum length of 500 bp, and CheckM2 was used to assess the quality of the reassembled bins. Bins that could not be reassembled due to an invalid k-mer coverage histogram error during assembly were ignored in the analysis.

### Reproducibility

We evaluated the run time and peak memory usage of the binning tools on a Cascade Lake 6242 machine equipped with an NVIDIA Quadro RTX 5000 GPU, using 1 CPU with 24 cores and 128 GB of main memory. To ensure reproducibility, we provide Snakemake workflows for coassembly multi-sample and multi-sample binning. These workflows cover the entire pipeline, including assembly, read mapping, alignment sorting, abundance matrix generation, and binning (Supplementary Figs 9 and 10). We have also developed a reassembly workflow that includes read recruitment for each bin, reassembly, scaffold filtering, and quality assessment using CheckM2 (Supplementary Fig. 11).

**Key Points**

- SemiBin2 and COMEBin, contrastive learning-based binners, yield the highest binning accuracy despite not consistently achieving the best embedding accuracy.
- Post-binning reassembly significantly improves the quality of low-coverage bins.
- Coassembly with multi-sample abundance is more effective for low-coverage data, while sample-wise assembly with multi-sample abundance performs better for high-coverage data.
- In multi-sample binning, clustering contigs separately within each sample’s embedding space improves performance over clustering first and splitting bins later.

## Supplementary Material

Supplementary_materials_bbaf617(1)

## Data Availability

Software, scripts used for data analysis, and summary results are available as open source at https://github.com/soedinglab/binning_benchmarking.git. Command lines for running the binning tools and parameters are given in the supplementary material. The datasets used from the CAMI2 study are available at https://frl.publisso.de/data/frl:6425521.
