# Supplementary material for: Evaluation of metagenome binning: advances and challenges
Source: Brief Bioinform. 2025 Nov 21;26(6):bbaf617. doi: 10.1093/bib/bbaf617 (PMC12636519; doi:10.1093/bib/bbaf617)
Supplement: Supplementary_materials_bbaf617(1) [file supplementary_materials_bbaf617(1).pdf]

Supplementary materials for Evaluation of Metagenome Binning: Advances and Challenges

Arangasamy Yazhini[1,*], Étienne Morice[1,2], Annika Jochheim[1,2], Benjamin Lieser[1], Johannes Soeding[1,3,*]

[1] Quantitative and Computational Biology, Max-Planck Institute for Multidisciplinary Sciences, 37077 Göttingen, Germany.

[2] International Max-Planck Research School for Genome Sciences, University of Göttingen, 37077 Göttingen, Germany.

[3] Campus Institute Data Science (CIDAS), University of Göttingen, 37077 Göttingen, Germany.

*Correspondence: yazhini@mpinat.mpg.de, soeding@mpinat.mpg.de

Comparison of the AMBER and CheckM2 evaluations

To evaluate the reliability of CheckM2 [1], we compared its completeness and contamination estimates to those calculated by AMBER [2], using binning results based on gold-standard contigs from the CAMI2 datasets. Supplementary Fig. 2 shows the comparison of the number of high-quality bins (90% completeness and 5% contamination), medium-quality bins (70% completeness and 10% contamination) and low-quality bins (50% completeness and 10% contamination). Overall, the results indicate that AMBER and CheckM2 produce comparable evaluations, and the relative performance rankings of the binners observed with CheckM2 are consistent with those obtained using AMBER.

We also compared the completeness and contamination values obtained for individual bins from AMBER and CheckM2. Supplementary Fig. 3 shows that CheckM2's completeness prediction correlates strongly with AMBER's prediction, with a Pearson correlation coefficient of 0.96. For the contamination measure, the Pearson correlation coefficient is 0.52, indicating a positive correlation, though not as strong as for completeness. The positive correlations suggest that the CheckM2 evaluation is a reliable benchmark for assessing binning performance on real datasets.

Commands used to run metagenome binning tools

```
#These commands were used for coassembly multi-sample binning
/usr/bin/time -v vamb --outdir vamb_results --fasta
↪  single_pooled_final.contigs.fa --bamfiles bamfiles/*_sorted.bam -m 1000
↪  --minfasta 200000 --cuda -p 24

/usr/bin/time -v SemiBin2 generate_sequence_features_single -i
↪  single_pooled_final.contigs.fa -b bamfiles/*_sorted.bam -o semibin2_results
↪  --min-len 1000 -p 24

/usr/bin/time -v SemiBin2 train_self --data semibin2_results/data.csv
↪  --data-split semibin2_results/data_split.csv -o semibin2_results -p 24

/usr/bin/time -v SemiBin2 bin_short -i single_pooled_final.contigs.fa --model
↪  semibin2_results/model.pt --data semibin2_results/data.csv -o
↪  semibin2_results/bins --minfasta-kbs 200 --min-len 1000 -p 24

/usr/bin/time -v run_comebin.sh -a single_pooled_final.contigs.fa -o
↪  comebin_results -p bamfiles -t 24

/usr/bin/time -v genomeface -i sorted_pooled_final.contigs.fa -o
↪  genomeface_results -a abundances_gf_sorted.tsv -g marker_hits -m 1000
```

```
/usr/bin/time -v vamb bin taxvamb --fasta sorted_pooled_final.contigs.fa --outdir
↪  taxvamb_results --bamfiles bamfiles/*_sorted.bam --taxonomy
↪  metabuli_results/taxonomy_result.tsv -m 1000 --minfasta 200000 --cuda -p 24

/usr/bin/time -v vamb bin taxvamb --fasta sorted_pooled_final.contigs.fa --outdir
↪  taxvamb_results_notaxometer --bamfiles bamfiles/*_sorted.bam --taxonomy
↪  metabuli_results/taxonomy_result.tsv -m 1000 --minfasta 200000 --cuda -p 24
↪  --no_predictor

/usr/bin/time -v metabat2 -i sorted_pooled_final.contigs.fa -a
↪  abundances_gf_sorted.tsv -o metabat2_results/metabat2_results -t 24 -m 1500
```

Supplementary Table

Table 1: Binning accuracy of deep-learning binners using either their own clustering algorithm or COMEBin's clustering algorithm. Increases in accuracy achieved through COMEBin clustering are highlighted in bold.

Binning accuracy	Marine		Strain-madness		Plant-associated	
	own	COMEBin	own	COMEBin	own	COMEBin
VAMB	0.55	**0.67**	0.22	**0.28**	0.24	**0.35**
SemiBin2	0.68	0.64	0.40	0.35	0.40	**0.45**
COMEBin	0.69	0.69	0.42	0.42	0.40	0.40
GenomeFace	0.63	0.56	0.48	0.31	0.35	**0.39**
TaxVAMB	0.67	0.67	0.18	**0.35**	0.28	**0.35**
TaxVAMB_wo_taxometer	0.65	0.66	0.19	**0.25**	0.28	**0.34**

Supplementary Figures

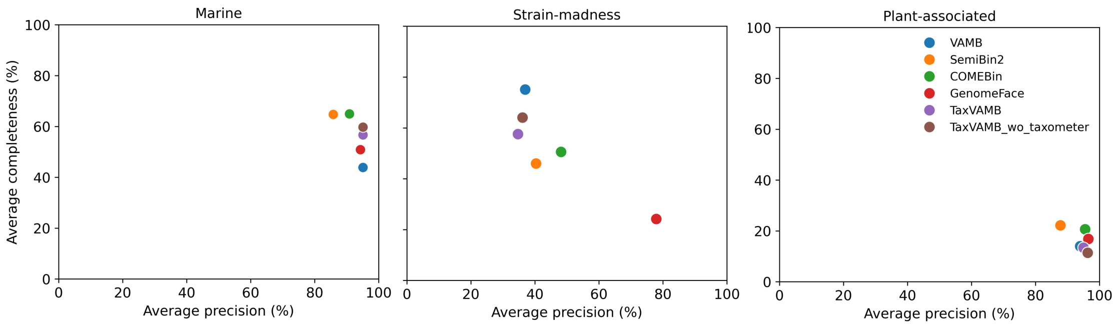

Figure 1: Average purity (bp) and average completeness (bp) of metagenomic bins generated by deep learning binners.

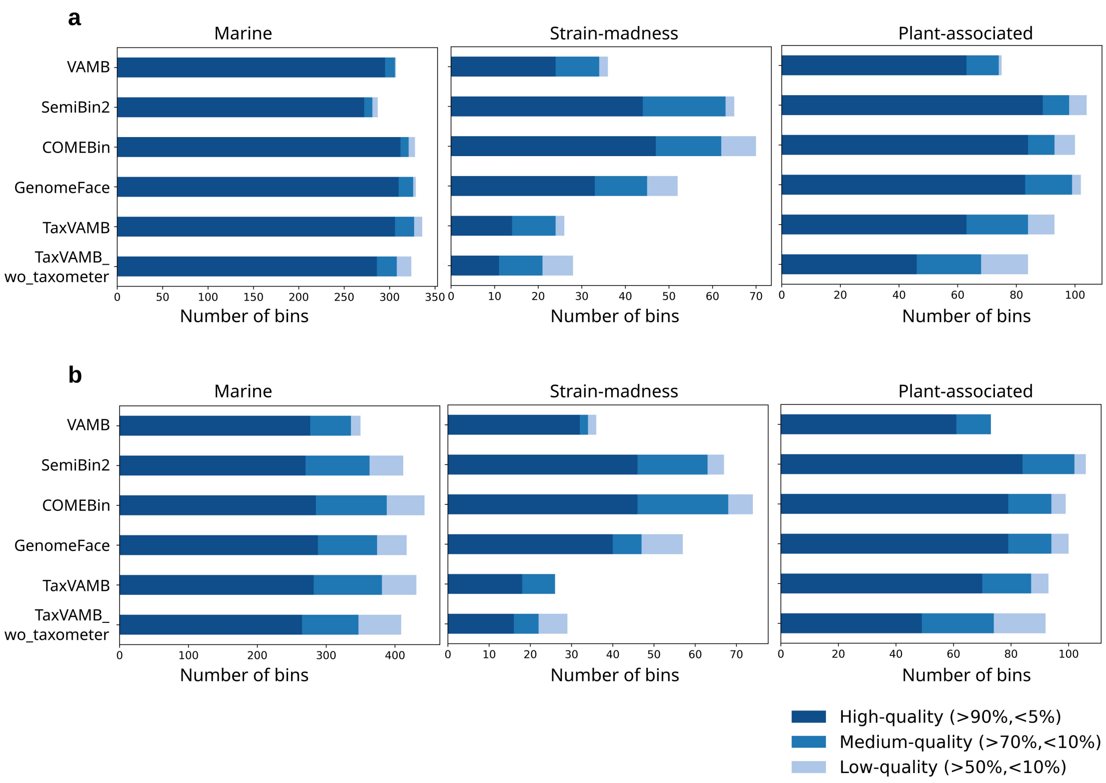

Figure 2: **Evaluation of deep-learning binners on coassembly multi-sample binning**. Plots show the number of high, medium and low quality bins based on completeness and purity computed using **a**) AMBER and **b**) CheckM2.

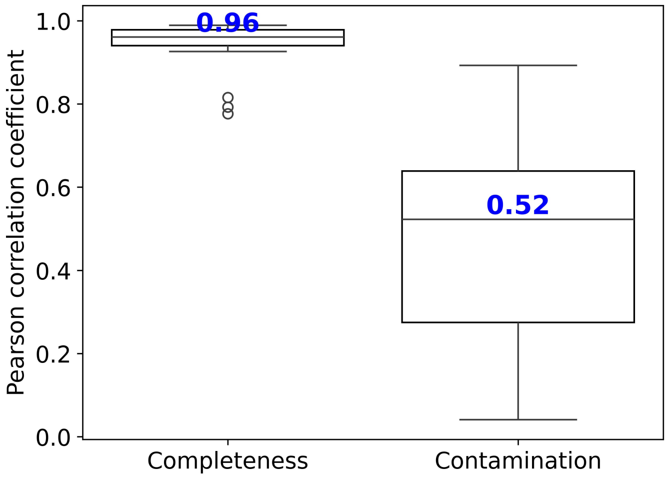

Figure 3: Pearson correlation coefficient between AMBER and CheckM2 predictions of completeness and contamination.

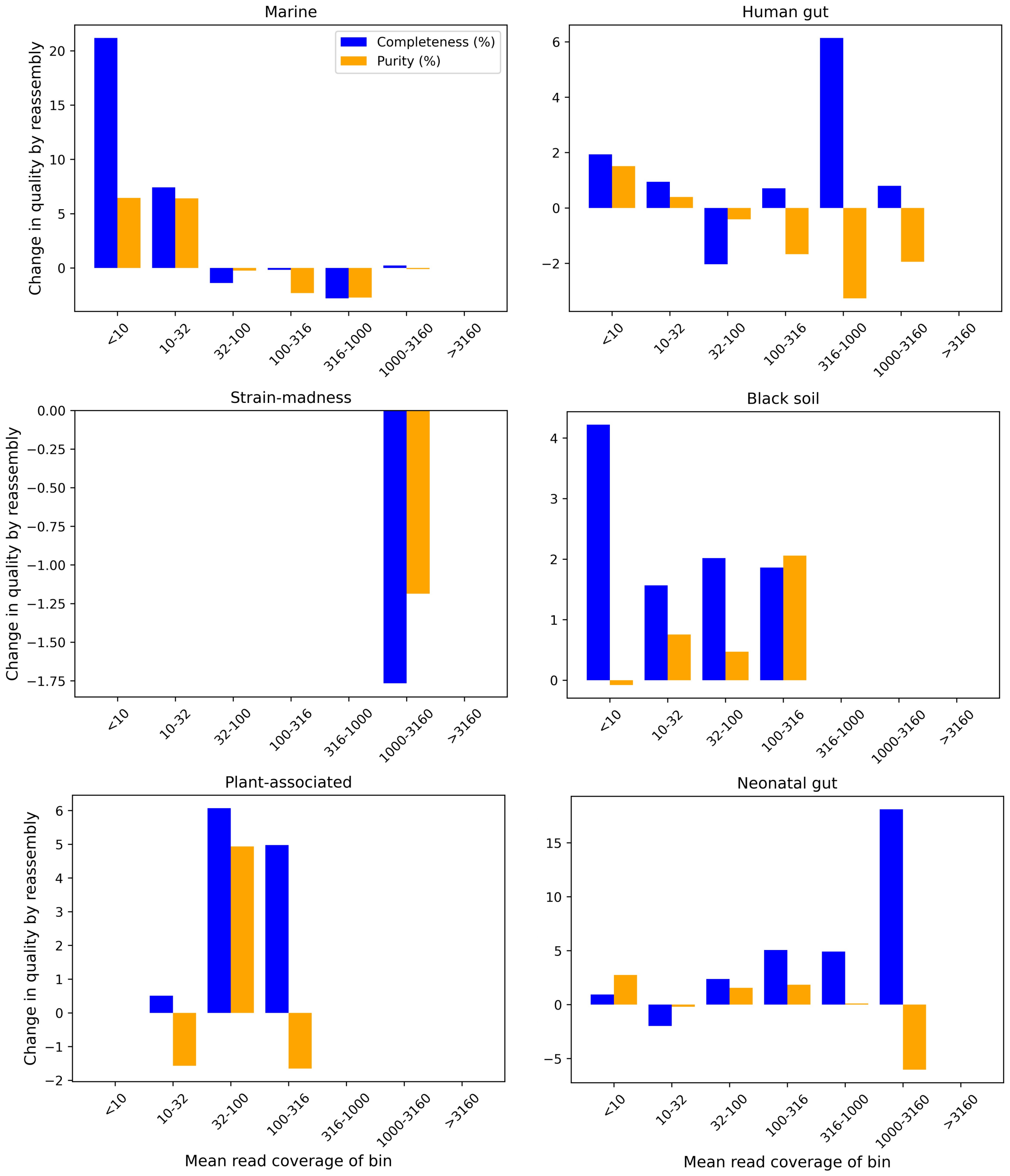

Figure 4: **Change in completeness (%) and purity (%) of bins upon reassembly**. X axis is the mean of total read coverage of contigs in a bin. Y axis is the difference in values (completeness after reassembly - completeness of bins before reassembly weighted by bin size and normalized by $1e^6$). Positive y axis values indicate improvement in quality values after reassembly. Bin assignment from GenomeFace's results was used for this plot.

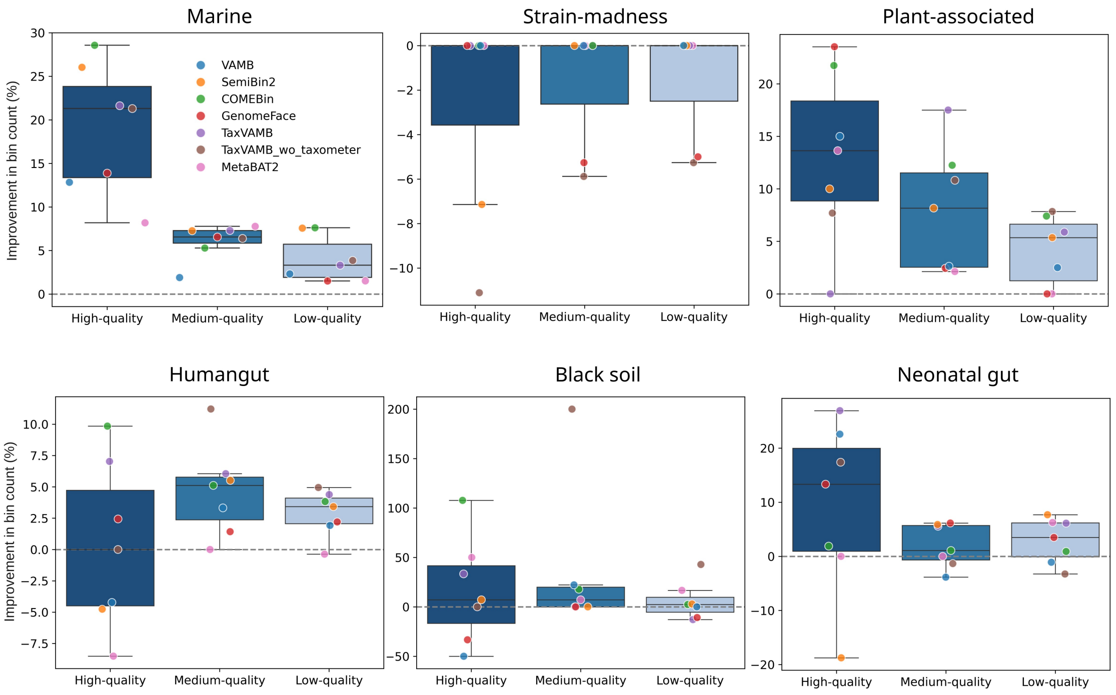

Figure 5: **Evaluation of reassembly after binning.** The percentage increase in the number of bins after reassembly belonging to high, medium and low-quality categories. The percentage increase in the number of bins for each binner is shown as dots.

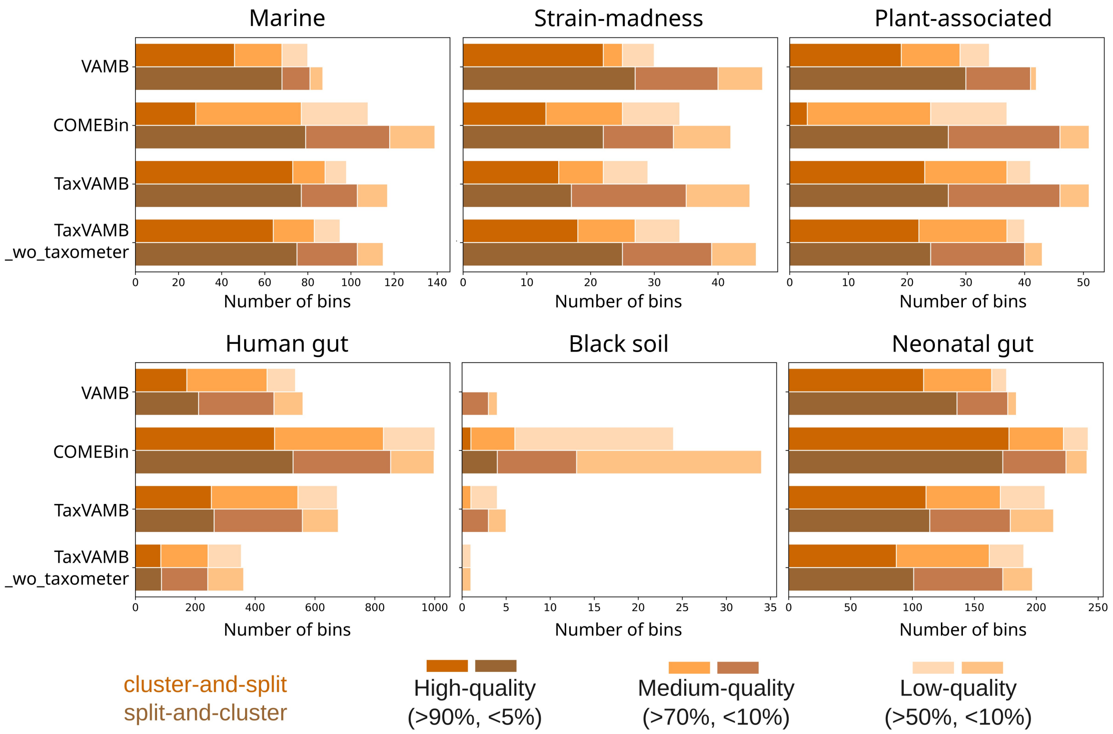

Figure 6: **Cluster-and-split *vs* split-and-cluster mode for multi-sample binning**. The number of non-redundant high, medium and low-quality bins obtained from two modes using the iterative density-clustering algorithm (employed by VAMB and TaxVAMB) and the iterative Leiden community detection algorithm (employed by COMEBin).

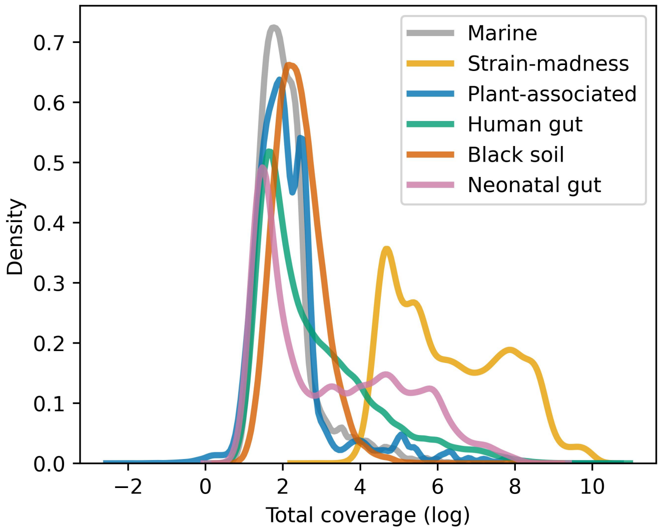

Figure 7: Density distribution of total read coverage of contigs binned in each dataset.

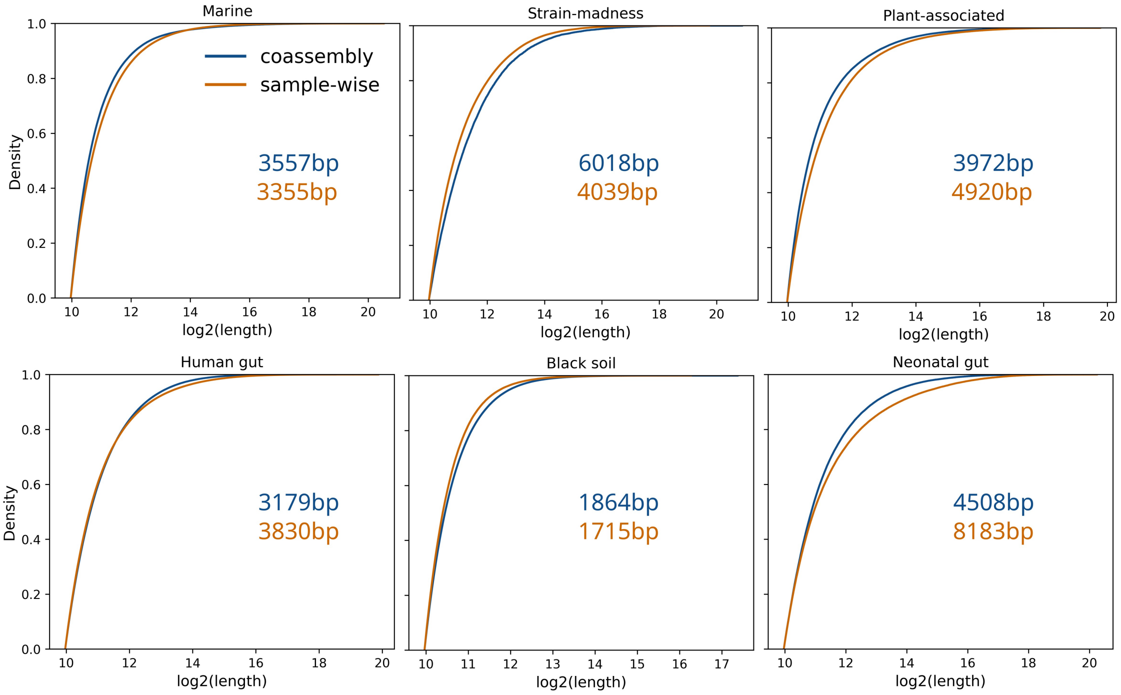

Figure 8: Cumulative density distribution of sequence length (base pairs in log scale) of contigs (minimum length $\geq$ 1000 bp) assembled by coassembly (blue) and sample-wise assembly (orange). Numbers written are the mean over contig lengths from different assembly approaches.

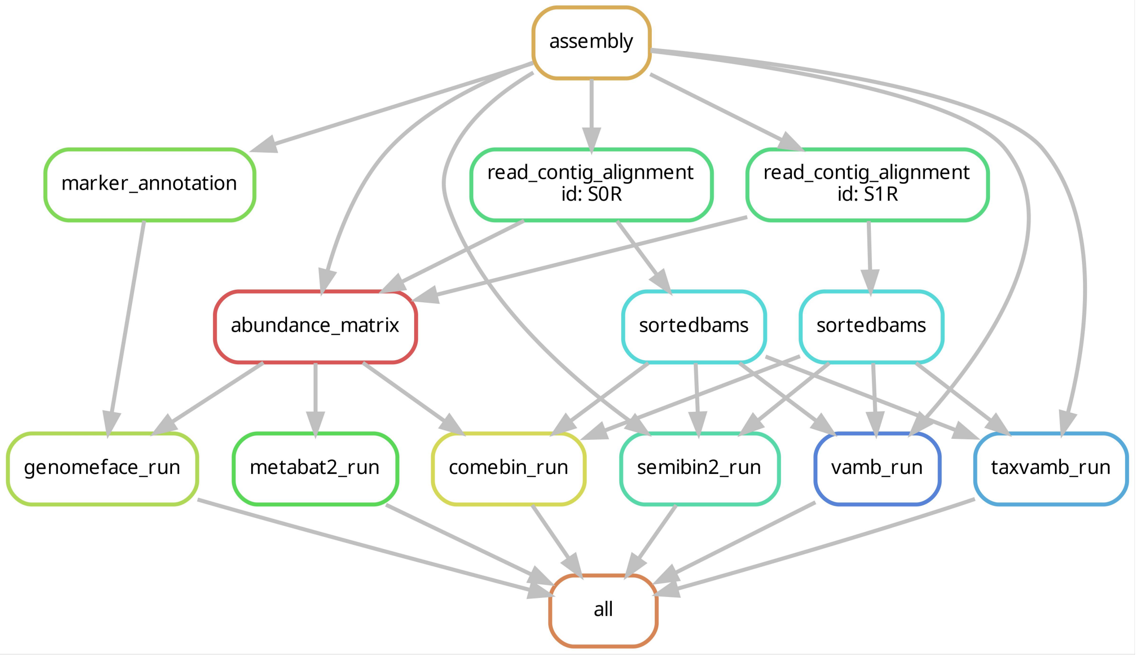

Figure 9: **Workflow for benchmarking coassembly multi-sample binning.** It uses MEGAHIT [3] for assembly, Strobealign [4] for read mapping, samtools for alignment sorting, and VAMB, SemiBin2, COMEBin, GenomeFace, TaxVAMB and MetaBAT2 for binning. The arrow indicates that the output of the previous step is used directly as input for the next step.

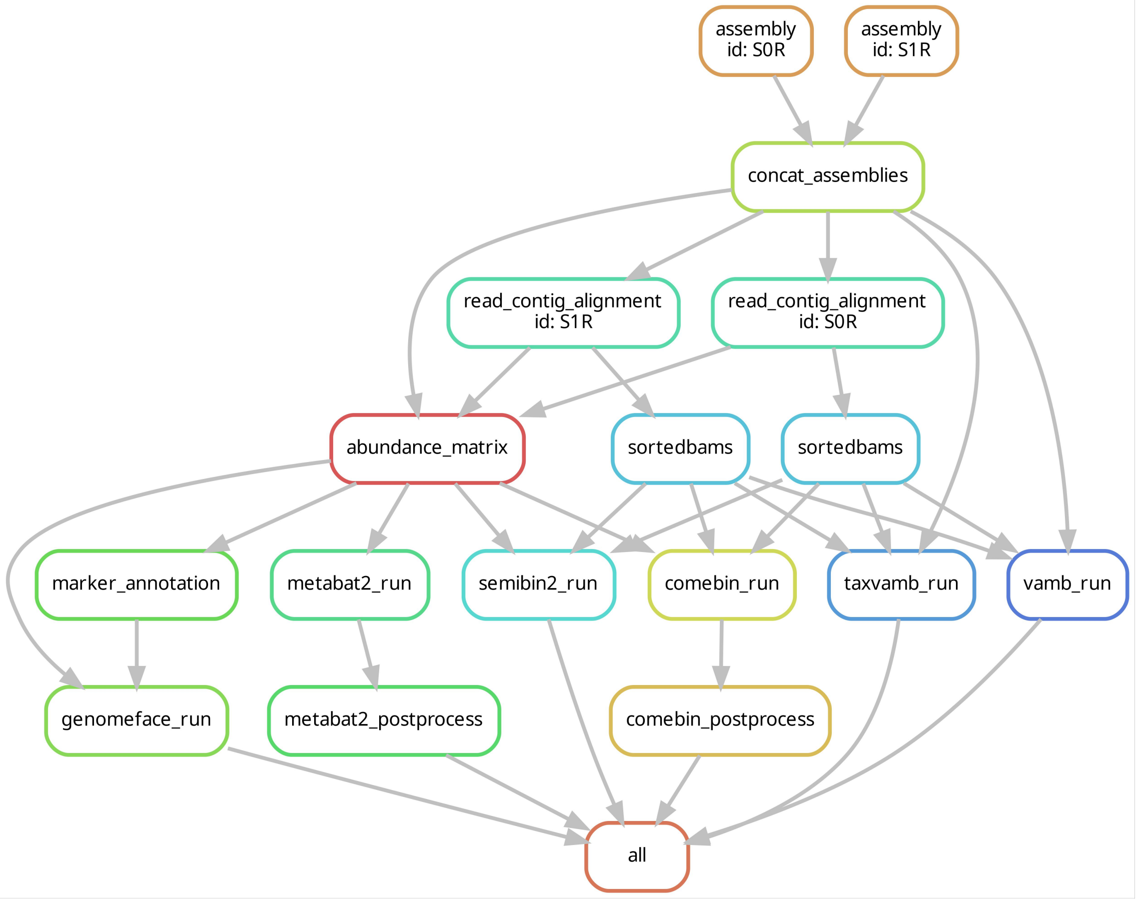

Figure 10: **Workflow for benchmarking multi-sample binning.** It includes assembly, read mapping, alignment sorting, binning and bin splitting by sample. The tools for each step are the same as those mentioned in Supplementary Fig.9.

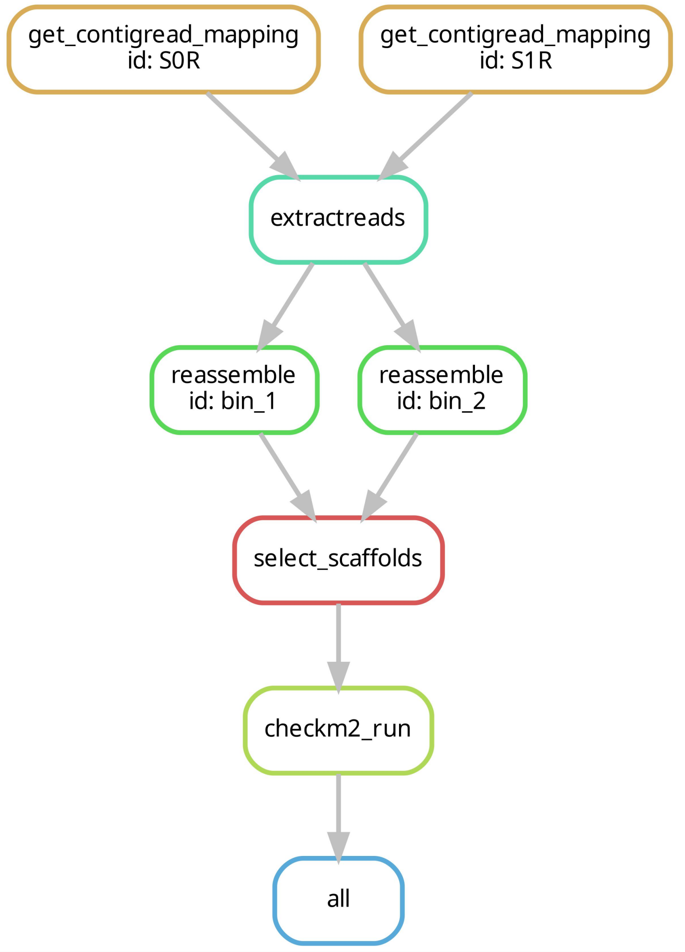

Figure 11: **Workflow for post-binning reassembly.** It recruits reads mapped to contigs belonging to a bin using our `extractreads` script, reassembles using SPAdes [5], filters scaffolds to produce final bins using `convertfasta_multi2single` script, and evaluates them using CheckM2 [1]. Scripts are available at *https://github.com/soedinglab/binning_benchmarking*.

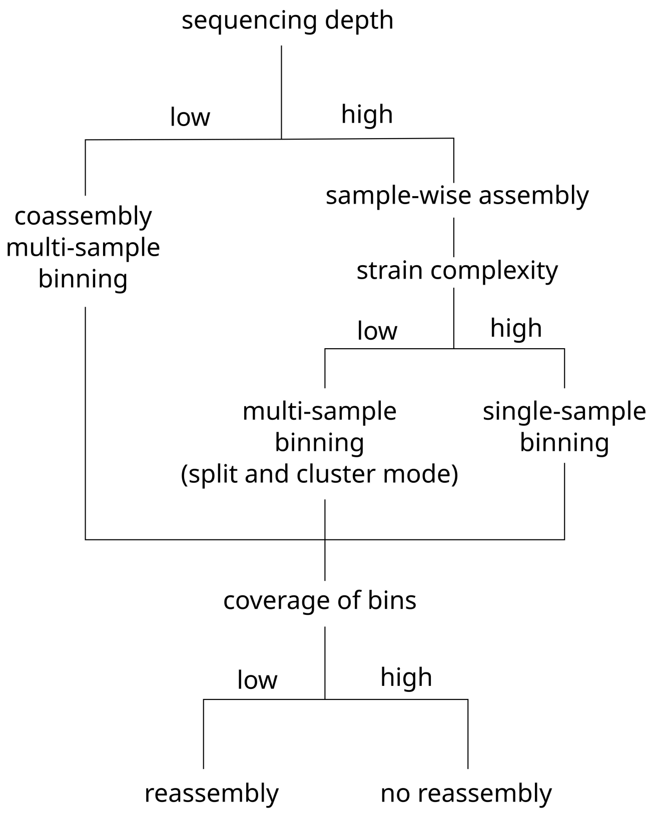

Figure 12: **Effective strategies for metagenome binning.** The flowchart outlines recommended strategies based on sequencing depth, strain complexity, and bin coverage.

References

[1] Chklovski, A., Parks, D. H., Woodcroft, B. J. & Tyson, G. W. Checkm2: a rapid, scalable and accurate tool for assessing microbial genome quality using machine learning. *Nature Methods* **20**, 1203–1212 (2023).

[2] Meyer, F. *et al.* Amber: assessment of metagenome binners. *Gigascience* **7**, giy069 (2018).

[3] Li, D., Liu, C.-M., Luo, R., Sadakane, K. & Lam, T.-W. Megahit: an ultra-fast single-node solution for large and complex metagenomics assembly via succinct de bruijn graph. *Bioinformatics* **31**, 1674–1676 (2015).

[4] Sahlin, K. Strobealign: flexible seed size enables ultra-fast and accurate read alignment. *Genome Biology* **23**, 260 (2022).

[5] Bankevich, A. *et al.* Spades: a new genome assembly algorithm and its applications to single-cell sequencing. *Journal of computational biology* **19**, 455–477 (2012).
